# Recent Progress in Long-Term Sleep Monitoring Technology

**DOI:** 10.3390/bios13030395

**Published:** 2023-03-17

**Authors:** Jiaju Yin, Jiandong Xu, Tian-Ling Ren

**Affiliations:** 1 School of Integrated Circuits, Tsinghua University, Beijing 100084, China; 2 Beijing National Research Center for Information Science and Technology (BNRist), Tsinghua University, Beijing 100084, China; 3 Center for Flexible Electronics Technology, Tsinghua University, Beijing 100084, China

**Keywords:** biosensors, sleep monitoring, polysomnography, REM sleep

## Abstract

Sleep is an essential physiological activity, accounting for about one-third of our lives, which significantly impacts our memory, mood, health, and children’s growth. Especially after the COVID-19 epidemic, sleep health issues have attracted more attention. In recent years, with the development of wearable electronic devices, there have been more and more studies, products, or solutions related to sleep monitoring. Many mature technologies, such as polysomnography, have been applied to clinical practice. However, it is urgent to develop wearable or non-contacting electronic devices suitable for household continuous sleep monitoring. This paper first introduces the basic knowledge of sleep and the significance of sleep monitoring. Then, according to the types of physiological signals monitored, this paper describes the research progress of bioelectrical signals, biomechanical signals, and biochemical signals used for sleep monitoring. However, it is not ideal to monitor the sleep quality for the whole night based on only one signal. Therefore, this paper reviews the research on multi-signal monitoring and introduces systematic sleep monitoring schemes. Finally, a conclusion and discussion of sleep monitoring are presented to propose potential future directions and prospects for sleep monitoring.

## 1. Introduction

### 1.1. Sleep

**Figure 1:**
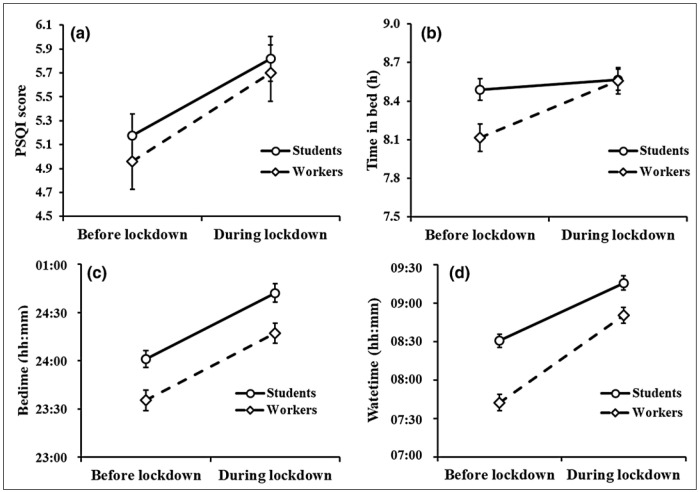
Changes in (**a**) Pittsburg Sleep Quality Index (PSQI) total score, (**b**) time in bed, (**c**) bedtime, and (**d**) wake time as a function of the presence of the lockdown and the status (worker or student) of the participants [9]. Reproduced with permission, 2020 European Sleep Research.

Sleep takes up about one-third of our lives. As shown in Figure 1, the COVID-19 outbreak has affected people’s sleep in many ways [1,2,3,4,5]. In the wake of the COVID-19 outbreak, it has been reported that many people’s sleep duration has increased, but at the same time, the sleep quality has declined, and the sleep time has changed [1]. Statistics have shown that 18.2% of people have poor sleep quality [2]. There was a general increase in the impact of sleep deficits and mental health burdens on healthcare workers. Sleep deprivation has increased prevalence in patients with acute and long-term COVID-19. Dreams under the epidemic [6] and post-vaccine effects [4] also impact sleep.

We should pay more attention to how people can achieve good quality sleep, including restful sleep, no daytime sleepiness, and adequate objective sleep depth [7]. Sleep duration and quality are the core indicators to evaluate whether a person has healthy sleep. Where sleep duration is easier to measure, evaluating sleep quality needs to find an easier metric. The microstructural sleep analysis of the cyclic alternating pattern may be related to self-reported sleep quality, that is, the measurement of the total duration of sleep and the analysis of sleep cycles are normally the most important for analyzing sleep quality (this will be specified in Section 2.2) [8].

### 1.2. Sleep Problems

Inadequate, irregular, or poor-quality sleep is common in modern society. Factors contributing to sleep deprivation include occupation, social demands, mental illness, physical illness, sleep disorders, race, age, marital status, gender, and hospitalization [10,11]. Sleep deprivation or sleep disorders can lead to low cognition, poor alertness, poor mood, cardiovascular disease, diabetes, metabolic and immune disorders, and even death [12,13].

For particular groups, sleep problems also have their own unique manifestations. Adolescents tend to sleep late, wake up early during school days, and catch up on sleep on weekends [14], leading to differences in their sleep on weekdays and days off [14,15]. Some older adults also experience sleep disturbances because the circadian system and sleep balance mechanisms become less robust with normal aging [16]. Finally, women with severe premenstrual syndrome (PMS) have poorer sleep quality, which may be related to altered melatonin rhythms [17]. There are also a variety of sleep disorders that may affect patients’ quality of life, such as obstructive sleep apnea, chronic insomnia, narcolepsy, delayed sleep–wake phase disorder, and Kleine–Levin syndrome [3].

For people with neurological and metabolic disorders, sleep quality is critical to health and even life. A classic example is people with depression. Antidepressant medications may affect sleep structure. Persistent sleep problems can, in turn, increase depression relapse or increased drug dependence and even potentially cause suicide in patients [18,19]. Attention to sleep problems can help determine the best medication regimen for depressed patients. In addition, for pregnant women, clinical pregnancy and live birth have occurred in 35% of women with sleep-disordered breathing (SDB) compared to 58% of women without SDB [20]. Sleep impairment is also a common comorbid and debilitating symptom for persons with opioid use disorder (OUD). Research into underlying mechanisms and efficacious treatment interventions for OUD-related sleep problems requires both precise and physiologic measurements of sleep-related outcomes and impairment [21].

### 1.3. Summary

Sleep is a complex physiological behavior, and the physiological signals and sensing techniques associated with sleep are diverse. Sleep problems are very common in the current society. Sleep monitoring technology is also very rich. However, at present, the most accurate monitoring system is for clinical use (in Section 2.1), which is difficult to use in daily life. There is a lot of research space for the technology that allows people to detect long-term sleep at home. This article will start with a brief introduction to professional polysomnography and its limitations. The main part focuses more on how various physiological signals can be monitored in the home. For monitoring that cannot be domesticated at the moment but is of great value, brief introductions and outlook are provided (mainly in Section 3.4, Section 4.7 and Section 5).

Section 2 introduces the standard clinical sleep monitoring technique, followed by the main focus of sleep monitoring: sleep cycles and sleep disorders. Section 3, Section 4 and Section 5 specifically summarize the work related to sleep monitoring. In this paper, we classify the relevant studies into three chapters based on the type of physiological signals collected. Section 3, Section 4 and Section 5 introduce bioelectrical, biomechanical, and biochemical signal monitoring, respectively. The classification is based on the type of signal generated by the body rather than the sensor output. For example, strain gauges are classified as biomechanical signal monitoring because they convert the body’s strain into an electrical signal. Optical sensors, which analyze blood flow rate by detecting reflected light, are also classified as biomechanical signal monitoring; using the same optical sensors to detect oxygen levels in the blood is classified as biochemical signal monitoring. Multi-signal monitoring is summarized in Section 6. Section 7 provides conclusions and discussion.

## 2. Sleep Monitoring

### 2.1. Polysomnography

**Figure 2:**
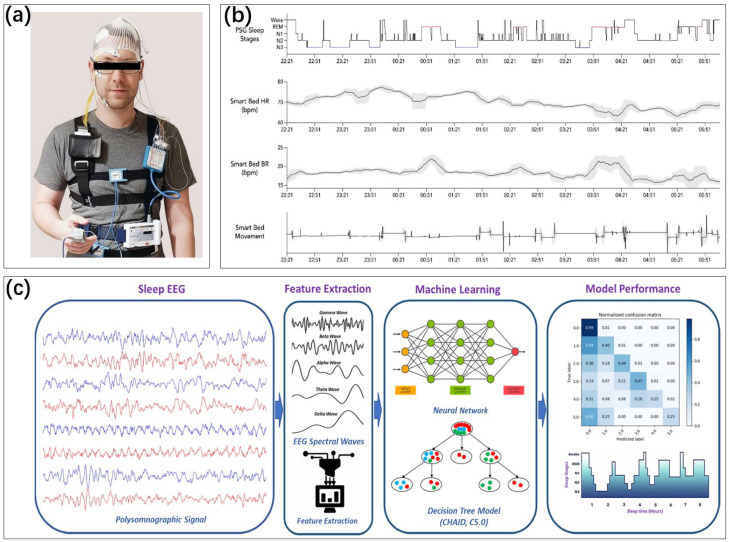
(**a**) The use of PSG [32,33]. Reproduced under the terms of the CC-BY Creative Commons Attribution License, Copyright 2022 by the authors, published by FRONTIERS MEDIA SA. (**b**) A home PSG that divides the sleep stages by monitoring several pieces of physiological information. Heart rate, respiratory rate, and movement are recorded and compared with the sleep cycle of a professional PSG [32,34]. Reproduced under the terms of the CC-BY Creative Commons Attribution License, Copyright 2022 by the authors, published by MDPI. (**c**) The principle of advanced polysomnography for sleep detection. Multichannel signals in PSG, after feature extraction, are analyzed using a machine-learning trained model. Sleep stage classification is performed [35]. Reproduced under the terms of the CC-BY Creative Commons Attribution License, Copyright 2022 by the authors, published by MDPI.

At present, the technology of sleep monitoring in the clinic is mature and abundant. A sleep monitoring technology that combines a variety of common sensing methods is called polysomnography (PSG). Standard PSG includes an electroencephalogram (EEG), electrocardiography (ECG), electrooculogram (EOG), and recordings of airflow, respiratory effort, oxygen saturation, and limb electromyography (EMG) [22]. These signals are collected and recorded simultaneously. PSG can detect the occurrence of sleep apnea (SA) or performing sleep stages (Figure 2b,c) [23]. PSG is widely used in hospitals for sleep monitoring. For example, in the intensive care unit (ICU), where special care of the patient is required, very comprehensive monitoring is performed. Methods for assessing and monitoring sleep in the ICU include polysomnography, bispectral indices, behavior charts, nursing assessments, and patient questionnaires [24]. However, technology that allows for long-term sleep detection at home is still necessary. This is for four main reasons.

First, autonomic adaptation processes within the central nervous system are significantly vulnerable when subjects sleep in a sleep laboratory [25]. The test results in the laboratory may not be representative of the state of everyday life. Since it is not convenient for home use, and people are not conscious during sleep, they may not be aware that they are suffering from sleep-related diseases in time. Obstructive sleep apnea (OSA) affects more than 900 million adults globally and can create serious health complications when untreated, while 80% of cases remain undiagnosed [26].

Second, sleep activity is inherently closely related to daytime life and a person’s overall level of health. So, it is not related only to the hospital but to their bedrooms and to their lives in general. Scientists have proven that there is a negative correlation between the number of steps taken for exercise and the onset of sleep apnea. So, sleep monitoring should be part of a complete, daily health test to help most people improve their sleep [27,28,29]. Figure 2a demonstrates the current use of polysomnography, which is not convenient in-home conditions. In the future, well-designed assays using new sleep measures or multimodal mobile wearable devices to assess the three domains of sleep and performance (objective sleep physiology, objective sleep quality, and subjective sleep quality) are needed to assess sleep status better and help people to improve their sleep.

Third, patients with poorer socioeconomic status may have lower odds of receiving good treatment due to cost and time occupation. Low socioeconomic status and its indicators (income, education, occupation, and employment) negatively correlate with PSG parameters [30]. Disappointingly, existing home-available sleep monitoring techniques yield sleep quality evaluations that do not correlate well with the subjective sleep perception of the user [31].

Finally, nighttime is when many sudden illnesses, such as sudden death, occur. Sudden cardiac death and epilepsy are common causes of sudden death, and most of these sudden deaths occur at rest or during sleep, even in younger age groups [36,37]. OSA is a common sleep breathing disorder. It causes nocturnal hypoxemia, sleep rhythm disorders, etc. OSA is associated with increased cardiovascular and cerebrovascular morbidity and mortality, including sudden cardiac death (SCD) [38,39]. Real-time monitoring is important for preventing sudden cardiac death during sleep.

### 2.2. Sleep Cycle

Human sleep is a complex physiological behavior that is complicated to evaluate comprehensively. However, a common evaluation criterion is whether a night’s sleep is characterized by multiple complete and healthy sleep cycles.

**Figure 3:**
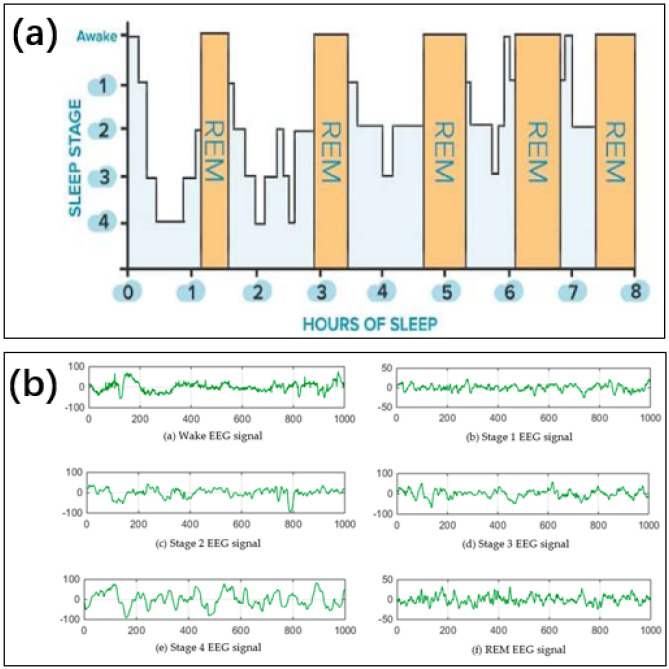
(**a**) Sleep cycles and REM sleep [41]. Reproduced under the terms of the CC-BY Creative Commons Attribution License, Copyright 2022 by the authors, published by MDPI. (**b**) EEG signals in different stages of sleep [42]. Reproduced under the terms of the CC-BY Creative Commons Attribution License, Copyright 2016 by the authors, published by MDPI.

Human consciousness can be divided into three states: wakefulness, non-rapid eye movement (NREM or non-REM) sleep (NREMS or non-REMS), and rapid eye movement (REM) sleep (REMS) [40]. NREMS can be further divided into three or four different stages. These stages alternate throughout the night, a phenomenon known as the sleep cycle. A cycle is roughly 2–3 h (Figure 3) [41]. Sleep with several complete sleep cycles is healthy.

The wakefulness period is the first stage of sleep when the person is still conscious. The EEG is a low-amplitude mixed-frequency signal with relatively high muscle tone, and the predominant EEG frequency is the alpha rhythm in the wakefulness period [40]. The eyes may move in response to the person’s consciousness.

**Figure 4:**
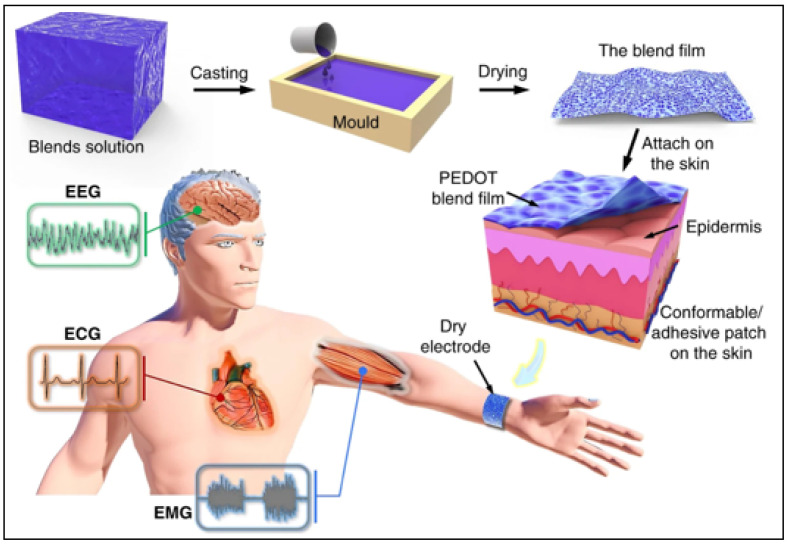
Fabrication and application of skin-surface electrode [58]. The resulting blend film can be used as an adhesive electrode on the skin for epidermal biopotential detections such as electrocardiography (ECG), electromyography (EMG), and electroencephalography (EEG). Reproduced under the terms of the CC-BY Creative Commons Attribution License, Copyright 2020 by the authors, published by Springer Nature.

When the alpha wave disappears, the person enters NREM sleep, in which the eye moves more slowly. This stage can be further subdivided into three stages according to the depth of sleep. The first one is when the person just enters the sleep state from the waking state when the sleep is very light and can be easily awakened. The second one is the longest and takes up about half of a person’s total sleep time. The third one is the deepest sleep, which has a large number of low-frequency delta waves in the brain waves [43]. This standard was published by the American Academy of Sleep Medicine (AASM) in 2007. The R&K criteria, widely used before that, was proposed in 1968 [44]. In the latter, using the slow wave percentage as a criterion, the stages of deep sleep are further split into S3 (20–50%) and S4 (50%). The difference can be clearly seen in the EEG images, so this classification method is still followed in many studies (in Figure 4) [42].

In the monitoring of the sleep cycle, REM is a very important stage. It accounts for 20–25% of nighttime sleep in healthy adults [45]. During this sleep stage, the brain is so excited that it is difficult to distinguish the EEG from waking hours while the muscles are most relaxed. This is why REM sleep is also called “paradoxical sleep” [43].

REM is considered to be the most unstable period of respiratory and cardiac sleep. Patients with diaphragmatic dysfunction may be particularly at risk due to the reduced tone of the accessory respiratory muscles. In addition, almost all antidepressants inhibit REM sleep [46]. The suppression of REM sleep in depressed patients may be one of the reasons for their poor sleep quality. A significant coupling of REM sleep cycles was observed when couples slept in the same bed. REM sleep may contain feedback to the surrounding environment [47]. Therefore, it is of great significance to monitor the physiological information during REM sleep.

The technique of performing the classification of sleep stages in clinical practice is well established. Awakening is with high muscle tone, targeted eye movements, and activated cerebral cortex. Non-REM sleep is characterized by moderate muscle tone, no eye movements, and slow EEG waves. REM sleep is characterized by low muscle tone, eye movements, and active cerebral cortex [43]. Thus, polysomnography is the standard gold method for measuring sleep cycles, but it is not convenient. As a result, the vast majority of patients do not receive effective diagnosis and treatment [48].

### 2.3. Sleep Disorders

The sleep disorders described in Section 1.2 and the sleep cycle abnormalities described in Section 2.2 are both problems with poor quality sleep itself. In addition to these, some sleep problems have additional manifestations. These are also important targets for sleep monitoring.

The first one is obstructive sleep apnea, which is a common sleep disorder. It is a blockage of the upper airway due to problems with sleep position, tongue position, etc. It may lead to problems such as low blood oxygen and interrupted sleep. Approximately 34% and 17% of middle-aged men and women, respectively, meet the diagnostic criteria for OSA. In contrast, there is a 40–80% prevalence among patients with cardiovascular disease [49]. OSA is one of the most regarded sleep disorders in sleep monitoring.

The second type is involuntary abnormal physical behavior during sleep. Restless legs syndrome is also one of the important sleep disorder disorders. About 10% of adults have experienced this condition that causes sleep disruption [50,51,52]. This abnormal behavior can affect a person’s quality of sleep and quality of life. Both OSA and restless legs syndrome can be monitored from multiple perspectives. Since there are different detection angles, such as EMG signal, motion, and heart rate (HR), they will appear several times in the text.

Sleep grinding, snoring, and nocturnal erectile dysfunction are also common disorders. However, because there are biomechanical signals that can correspond well, there are separate subsections for each in Section 4.

## 3. Bioelectrical Signal Monitoring

In polysomnography, multi-channel EEG signal detection and ECG signal detection are often clinically needed [23,53,54,55]. The simultaneous detection of eye movements with electrooculographic signals is important for monitoring REM sleep. The activity of the human trunk and extremities is also commonly measured by EMG signals. Bioelectric signals can be used for the monitoring of numerous physiological phenomena, and the measurement of bioelectric signals can be achieved by applying electrodes to the skin’s surface as shown in Figure 4 [56,57,58]. This non-invasive, inexpensive, and pervasive detection method has achieved large-scale applications.

However, the wires connected during bioelectric signal acquisition may cause a lot of inconvenience to the person. The need to ensure the effective fit of the electrodes also limits their use in daily life [59]. In recent years, new technologies such as wearable devices, electronic skin, and conductive fabrics have made wireless or even senseless bioelectric signal measurement possible [60,61]. The design at the device, circuit, and algorithm levels has allowed the measurement of wearable bioelectrical signals to be free from problems such as motion artifacts, facilitating the daily use of lay people and greatly expanding its application prospects [62]. In addition to the advancement of measurement technology, the development of theoretical research has also allowed more room for the application of bioelectrical signal measurement in sleep monitoring. More electrical signals related to sleep monitoring, such as electroretinography (ERG) [63], are being reported.

**Figure 5:**
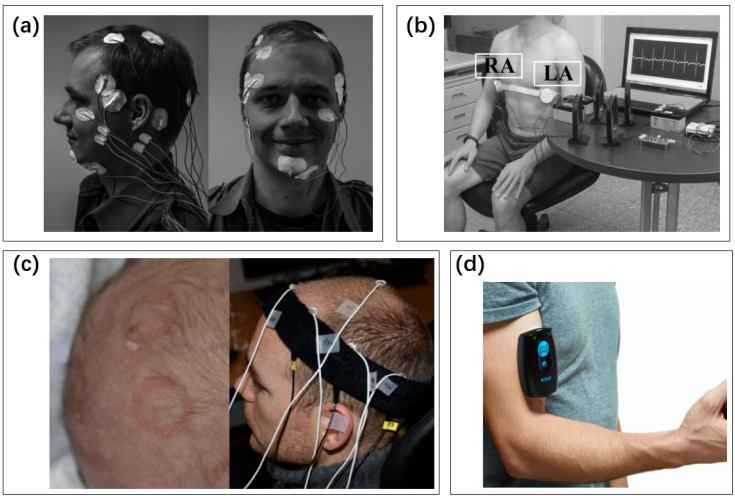
(**a**) Six EEG electrodes, two EOG electrodes, two chin EMG electrodes, two mastoidal reference electrodes, and bipolar EMG electrodes placed on the masseter [56]. Reproduced with permission, Copyright 2017 European Sleep Research Society. (**b**) Wearing of ECG [64]. RA and LA electrode pairs are placed between 5th and 6th ribs to avoid muscle movement interference. Reproduced under the terms of the CC-BY Creative Commons Attribution License, Copyright 2020 by the authors, published by MDPI. (**c**) Fabric electrodes are designed to avoid clicking on the patch from affecting the skin. The EEG sensor is fixed in the headband [65]. Reproduced under the terms of the CC-BY Creative Commons Attribution License, Copyright 2012 by the authors, published by MDPI. (**d**) EMG [66]. Reproduced with permission, Copyright 2018 International League Against Epilepsy.

This section is divided into a total of six subsections. The first four subsections introduce EEG, ECG, EMG (including EOG), and ERG separately, focusing on the significance and effect of monitoring. Figure 5 illustrates several typical schematic diagrams of bioelectrical signal detection. The electrode techniques used in several monitoring modalities will be summarized in Section 3.6. Section 3.5 is for passive bioelectrical detection.

### 3.1. Electroencephalography

EEG has long been an important part of sleep monitoring. The changes in brain waves during the various stages of the sleep state have been described in Section 2.2, and this has become a crucial item in sleep monitoring. Brain-wave characteristics are the gold standard for sleep cycle classification. The most accurate sleep staging analysis system is based on EEG, the only single sensing modality capable of identifying all sleep stages [67].

**Figure 6:**
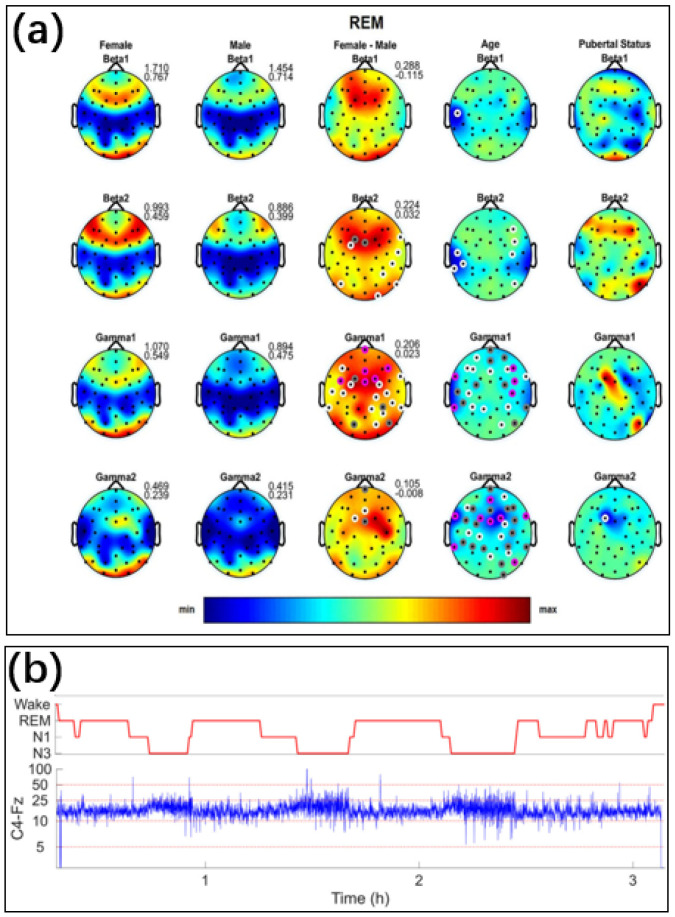
(**a**) Topographic distribution of absolute sleep EEG power (µV2) across derivations for the beta 1 to gamma two bands during REM sleep. The first and the second column depict the values averaged across females and males on the same scale, while the third column depicts the difference between females and males. Minimum and maximum values are shown in the upper right corner of each topographic map. The fourth and the fifth column show the F-values from the analysis of variance (ANOVA) for the factors of age and pubertal status. In the third to fifth columns, significant electrodes are shown in white (*p* < 0.05), gray (*p* < 0.01), and magenta (*p* < 0.001). In these columns, warm colors represent increased activity in females, while cool colors represent increased activity in males [69]. Reproduced under the terms of the CC-BY Creative Commons Attribution License, Copyright 2020 by the authors, published by Springer Nature. (**b**) EEG signals of C4-Fz channels under different sleep stages [70]. Reproduced under the CC-BY-NC-ND license, Copyright 2022 by International Federation of Clinical Neurophysiology, Published by Elsevier B.V.

The brain, as the most important nerve center in the body, has very distinct characteristics during all stages of the sleep cycle and has good results as a sleep monitoring indicator. As shown in Figure 6, the performance of brain waves varies greatly from stage to stage and between different genders [68,69,70], which is why the EEG has become the gold standard for sleep cycle identification. EEG can reflect the effects of previous nights of sleep, over-the-counter and prescription drugs, and even illicit drugs on brain activity during sleep [71]. In 2006, Guilleminault et al. studied the effects of different levels of sound stimulation on human brain waves during sleep and on the performance of sleepiness the next day, examining the analysis of the quality of disturbed sleep at the level of brain waves [72]. In addition to this, many studies have been reported on related detection devices due to the importance of EEG signals in the diagnosis of Alzheimer’s disease, Parkinson’s disease, epilepsy, etc. [73,74].

Miniaturization of traditional electrodes, or home use, allows for wearable EEG monitoring, but the principle remains that electrodes attached to the skin’s surface can pick up electrical signals of neural activity [56]. With the arrival of the new coronary epidemic, many people are less willing to go to the hospital, and home healthcare has become a healthcare trend. In 2020, Arnal et al. fabricated EEG sensors integrated into a headband. The mean percentage error of the EEG signal obtained with PSG monitoring α was 15 ± 3.5%, β was 16 ± 4.3%, λ was 16 ± 6.1%, and theta frequency during sleep was 10 ± 1.4% [75]. In 2021, Hsieh et al. developed a real-time EEG acquisition system for home use and used a deep-learning model that allowed the average absolute error of the wearable device to measure sleep efficiency to be reduced to 1.68% [76]. Studies using machine-learning algorithms for brain-wave recognition and analysis based on the same sensors are beyond the focus of this paper, but these studies are a good example of the significance of brain-wave sensors [77].

EEG is the most demanding for signal quality in bioelectrical signal sensing. Very often, there is a balance between wearing comfort and signal quality. Conventional EEG uses patch electrodes that have good signal quality but are not breathable and may cause skin swelling (see Figure 5c). To minimize the effects of contact resistance, Li et al. designed an array of microneedles that can be pierced into the skin and prepared the apparatus on a flexible substrate. The electrodes have record low skin–electrode contact resistance, 1/250th that of conventional electrodes.

From another improvement perspective, many researchers are exploring more user-friendly forms of wearable sensors. In 2019, Shustak et al. prepared soft, non-gel flexible electrodes with printed electrode technology to improve the comfort of brain-wave detection [61]. In 2017, Nakamura et al. designed the acquisition of brain-wave signals in the ear, which also achieved good results compared to the acquisition of brain-wave signals in the scalp patch [78]. In 2021, da Silva et al. designed flexible printed electrode sensors in the ear using graphene electrodes and combined them with a smartphone for recording and analysis [79].

Finally, in recent years, implantable brain–computer interfaces have enabled stable monitoring of signals [80,81,82,83]. Although its main application area is to assist people with motor impairments to control assistive devices [80,81], sensors on EEG signals may further advance the development of sleep monitoring technology in the future. Topchiy et al. studied in vivo implanted electrodes to monitor sleep in mice. They experimented with sleep monitoring with implanted electrodes and telemetry and found they could classify sleep stages more effectively than in vitro monitoring devices [84]. As the technology of implanted electrodes matures, this may also be a future technology that can strike a good balance between contact resistance and non-sensory use.

### 3.2. Electrocardiography

**Figure 7:**
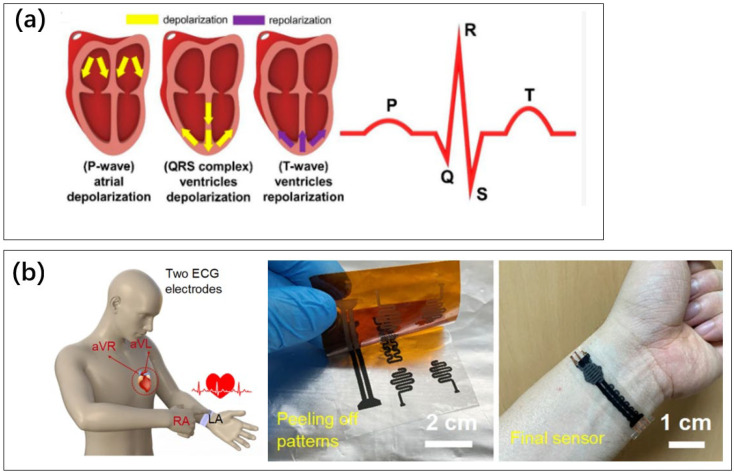
(**a**) The different phases of muscle movement during the heartbeat produce different parts of the ECG curve. The depolarization and repolarization of the heart for the generation of a P-wave, QRS complex, and T-wave [85]. Reproduced under the terms of the CC-BY Creative Commons Attribution License, Copyright 2022 by the authors, published by MDPI. (**b**) A flexible skin electrode sensor. Monitoring ECG at the wrist [90]. Reproduced under the Creative Commons Attribution 4.0 International License, Copyright 2022 by the authors, published by Nature Portfolio.

ECG is an important physiological examination closely related to sleep cycles and sleep apnea [85]. As shown in Figure 7a, the periodic movement of the heart will show different electrical signals and form regular ECG curves [85]. In hospitals, ECGs are collected through specialized equipment with the help of professional staff, but self-monitoring by patients is hardly up to this standard [86]. The fit of wearable device contacts is also a common problem. For this reason, many studies have expanded the relevant algorithms and databases so that testing devices can be adapted to self-testing using devices such as wearables to improve signal-to-noise ratios outside the hospital, exclude motion artifacts [87,88], and more accurately determine the occurrence of phenomena such as arrhythmias [89].

Wearable ECG allows the detection of sleep apnea. The classification accuracy obtained from the ECG belt has a sensitivity of 70% and a specificity of 74%, while the patched ECG has a sensitivity of 88% [91]. Single-lead ECG, worn on the abdomen, can also be good for detecting sleep apnea index and abnormal breathing [92]. In 2019, Hammour et al. studied in-ear ECG. The delay was reduced by up to 88% [93].

The electrodes can be kept naturally close to the skin compared to watch-type and headphone-type ECG measurement devices. ECGs on the torso often require patch electrodes, and many electrode materials can be irritating to the body. ECG sensing can be integrated into clothing and localized to locations with good signal-to-noise ratios [94] (Figure 7b).

Compared to EEG, ECG requires less signal quality, so many studies can use non-wearable, skin-tight electrodes. Lim et al. arranged electrodes on a mattress [95]. Won Kyu Lee et al. integrated flexible electrodes into the mattress to collect ECG signals from the skin’s surface after a person lies on it [62]. This avoids the need to wear a dedicated device and is well-suited for sleep scenarios. In 2020, Klum et al. used multimodal ECG and analyzed the effects of different sleeping positions [96]. Left ventricular ejection time and pre-ejection period estimation errors were 10% and 21%.

### 3.3. Electromyography and Electrooculography

**Figure 8:**
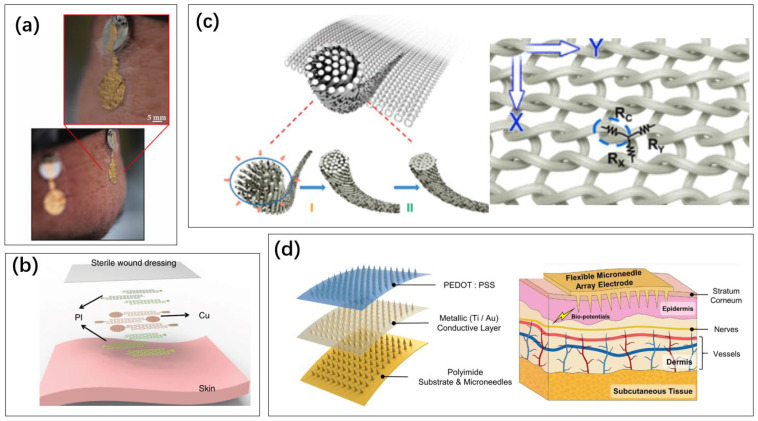
Method for collecting electrical signals on the skin’s surface: (**a**) gold electronic tattoo [99]. Reproduced under the terms of the CC-BY Creative Commons Attribution License, Copyright 2023 by the authors, published by MDPI. (**b**) Multi-layer electronic tattoo [100]. Reproduced under the terms of the CC-BY Creative Commons Attribution License, Copyright 2020 by the authors, published by Springer Nature. (**c**) Conductive fabrics doped with graphene [101]. Reproduced under the terms of the CC-BY Creative Commons Attribution License, Copyright 2022 American Chemical Society. (**d**) Microneedle array electrodes on a flexible substrate [102]. Reproduced under Creative Commons Attribution 4.0 International License, Copyright 2022 by the authors, published by Shanghai Jiao Tong University Press.

EMG can conveniently reflect human muscles’ tension and limb activity and assist in measuring sleep cycles. While the monitoring of limb movements is challenged by mechanical sensors or camera sensing (in Section 3), EMG has an irreplaceable role in many fields. For example, it is difficult to monitor eye movements outside of the body because the eyelids obscure them in the human sleep state; nocturnal muscle tensions, such as changes in neck muscle tone, do not manifest as obvious changes in limb position. On the other hand, such muscle behaviors have electrical signals that can penetrate the tissues and be measured on the skin’s surface with good accuracy [97,98]. A variety of new methods of collecting electrical signals on the skin’s surface in Figure 8 enable convenient daily measurements of EMG.

In 2007, Magosso et al. used electrooculography to assess the sleep cycle, which proved to be very reliable, addressing the high labor cost and inconsistency of previous manual scoring [103]. Eye movement is tracked by several muscles. Skin electrodes affixed to the corners of the eye can pick up electrical signals and thus determine whether eye movement is occurring. Beach et al. achieved comfortable wear of eye movement detection devices by integrating EOG sensors in an eye patch through fabric sensor electrodes made of nylon and graphene. Though, with EOG alone, the accuracy of sleep time calculation is only about 70% [104]. However, EOG can be included in the sleep cycle analysis as an important item in sleep polysomnography.

Iranzo et al. used polysomnography to analyze EMG analysis of REM sleep in patients with REM sleep behavior disorders. These patients need more accurate monitoring of their REM sleep. EMG of the cardiac, flexor superficial, and extensor profundus muscles can help in the identification of REM [105]. Maeda used single-channel EMG, which also enabled sleep mydriasis detection with 100% sensitivity and specificity under some conditions, demonstrating that single-channel EMG signals can also be of good monitoring value [106].

In 2018, Beniczky et al. used wearable EMG signals to capture the evolution of TCS-related signals on the human surface for the detection of muscle rigidity and epilepsy occurring during sleep [66]. In 2022, Yeung et al. completed the diagnosis of obstructive sleep through muscle electrical signals in the tongue and epiglottis to epiglottal pressure and nasal airflow and then through EMG at the level of muscle movement [107]. The diagnosis of apnea was made by Rebelo after collecting the EMG signals generated by the apical muscles of the tongue and generating electrical signals to stimulate the apical muscles of the tongue to terminate the respiratory obstruction when sleep apnea was detected [108]. In 2018, Yamaguchi et al. designed a wearable miniature EMG system weighing 9 g, including the battery, to assess the occurrence of nocturnal teeth grinding [109] (sleep bruxism is described in detail in Section 3.3). In 2019, Prasad et al. connected the EMG device to a smartphone and used it to assist in monitoring teething behavior [110].

### 3.4. Electroretinography

Among the various types of electrical signals, the study of retinal electrical signals was the latest to begin and has the least application in sleep monitoring. The retinal electrical signal expressed the perception of light by the retina and was first used for the diagnosis of eye diseases [111].

**Figure 9:**
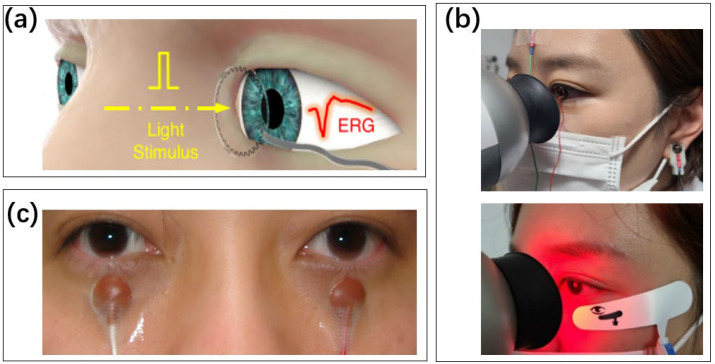
(**a**) Schematic diagram of ERG sensing [114]. The retina produces an electrical signal when light enters the human eye. A wire needs to be introduced to the surface of the eye to read the signal. Reproduced under the terms of the CC-BY Creative Commons Attribution License, Copyright 2021 by the authors, published by Springer Nature. (**b**) Conventional ERG monitoring requires leads from the surface of the eye, making it difficult to use for long periods of time. The new sensing method allows ERG signals to be collected on the skin around the eye, and all-night monitoring is possible [115]. Reproduced under the CC-BY-NC License, Copyright 2021 by the Korean Ophthalmological Society, published by Korean Ophthalmological Society. (**c**) A skin electrode was placed on the lower lid of each eye. The contralateral eye was not covered. A gold-cup electrode was placed on the right earlobe as the ground electrode [116]. Reproduced under the Creative Commons Attribution License, Copyright 2023 by the author(s), published by Public Library Science.

With the development of basic research demonstrating the influence of the light environment on human circadian rhythms, the response of the human nervous system to light became an item in sleep monitoring. In 1994, Galambos et al. found that ERG amplitude during slow-wave sleep was more than twice as high as during wakefulness. Moreover, ERG patterns during REM sleep were different from those during slow-wave sleep. Galambos confirmed that ERG signals are also associated with the sleep cycle [112]. In 2016, Liguori et al. demonstrated that ERGs could differ in patients with obstructive sleep apnea [63]. However, the relationship between fundus disease and sleep needs to be further explored [113] (Figure 9c).

Since ERG often requires electrodes placed on the inner eyelid (Figure 9a), it is more difficult and device-demanding to use than tests such as EOG, which can be applied to the skin. Research is also underway to attach electrodes to the skin around the eye to monitor ERG [115]. However, the signal quality is still not as good as the intraocular type. In addition, the need for ERG signals in sleep monitoring needs to be supported by more studies.

### 3.5. Passive Bioelectricity Detection

The electrical signals generated by the nervous system during activity are voltages actively generated by the body. The four previous vignettes are based on this. However, the human body can also be considered as a load consisting of resistance and capacitance, and passive bioelectrical detection is achieved by applying voltage and an electric field.

Blood pressure (BP) causes changes in the diameter of the blood vessels, which in turn affects the impedance of the tissues. Kireev et al. used graphene electronic skin to detect the impedance between electrodes at different locations on the skin [117]. The signal of impedance change can be detected as blood flow pulses move through the blood vessels. BP was calculated with an accuracy of 0.2 ± 4.5 mm Hg for diastolic pressures and 0.2 ± 5.8 mm Hg for systolic pressures.

Changes in human posture and position in the external electric field will lead to different polarization responses. By arranging the Wi-Fi device in the room after the placement design, the receiver can detect the human body’s activities. Epilepsy detection with 100% sensitivity is achieved without wearing a human device [118]. This method can also achieve 92% accuracy in the recognition of rhythmic movement disorders [119]. The use of multi-antenna arrays allows for the acquisition of richer electric field information. Yu et al. achieved 81.8% classification of sleep stages and breath detection with an average error of 0.23 bpm based on a multi-antenna Wi-Fi receiver [120].

In these two examples, mechanical changes in the human body cause changes in electrical properties. They are placed in Section 2, as the sensor collects electrical signals directly. More research will be reported in Section 3 on BP and motion.

### 3.6. Summary

There are similarities in the techniques used to acquire bioelectric signals on the skin’s surface. For example, electrodes used to monitor ECG might also be used to monitor EMG. However, different optimizations are needed in specific daily monitoring contexts.

In addition to the sensor itself, the monitoring object’s different back-end algorithm also greatly impacts the detection accuracy. For example, it is unfair to compare the accuracy of one sensor measuring EEG for sleep stage classification with the accuracy of another sensor measuring ECG for heart rate analysis. ECG has a strong regularity and can still measure heart rate with relative ease in the presence of noise interference. However, EEG is inherently more non-smooth and random, and its detection requires a higher signal-to-noise ratio. To focus on the effect of the sensor itself, the signal correlation of EEG acquisition was compared (with the standard Ag/AgCl wet electrode used clinically as a reference) [121].

Therefore, it is possible to compare the sensor electrodes that have appeared so far in Table 1.

## 4. Biomechanical Signal Monitoring

In the last section, electrical signals were reviewed. However, many human physiological behaviors and phenomena cannot be fully monitored by electrical signals at present, so the direct detection of mechanical signals in the human body is of irreplaceable significance.

In the absence of integrated dedicated health sensors, some smartphones determine the length of time a person sleeps based on the amount of time they are stationary [125]. This is one of the simplest ways to analyze sleep based on behavioral science, which is an important sleep monitoring item [126]. Nocturnal motor and nonmotor symptoms and other comorbid sleep disorders can disrupt sleep [127]. Diseases related to limb movement, such as Parkinson’s disease, are closely linked to sleep. This is a very primitive way of recording, but there is a big difference in how people behave during sleep and when awake.

This section focuses on sensing sleep-related mechanical signals, including posture, motion, acceleration, respiratory airflow, blood flow, etc.

### 4.1. Motion Detection

Limb movement is an important concomitant behavior during sleep; many people experience vigorous limb movement. Based on motion sensors on the wrist, Chun et al. monitored how often people with dermatitis may itch at night, demonstrating the effects of pruritus on sleep [128]. In addition to the common sleep onset tests and sleep stage divisions, some sleep disorders are also reflected in body movements. The most typical one is restless legs syndrome. In 2022, Brooks et al. used conductive fabric to form a capacitance with a person’s body, and the magnitude of this virtual capacitance changed after a change in the person’s posture, which in turn was detected. The potential improvement in diagnostic accuracy for assessing sleep disturbances associated with restless legs syndrome using this method can be estimated at approximately 68.1%, far exceeding the diagnosis of measuring anterior tibial EMG signals [129].

**Figure 10:**
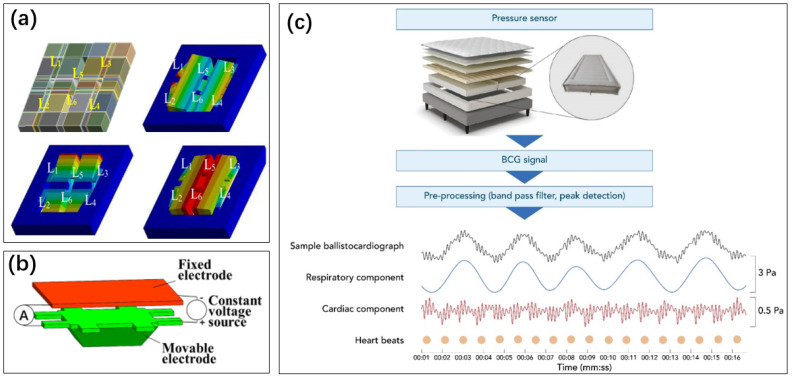
(**a**) Acceleration sensors made using MEMS technology, the mass block (6 L-beams L1 to L6) is displaced by the acceleration [133]. Reproduced under the terms of the CC-BY Creative Commons Attribution License, Copyright 2019 by the authors, published by MDPI. (**b**) The displacement of the object block brings about a change in capacitance [134]. Reproduced under the terms of the CC-BY Creative Commons Attribution License, Copyright 2020 by the authors, published by IOP Publishing Ltd. (**c**) A pressure sensor made of a multi-layer structure monitors the changes in received pressure [34]. Information such as human movement and heartbeat can be resolved. Reproduced under the terms of the CC-BY License, Copyright 2022 by the authors, published by MDPI.

Wristband motion sensors are the most common form of sleep monitoring. Accelerometers based on a micro-electro-mechanical system (MEMS) can be combined with everyday wearable items such as watches for wearability (Figure 10a,b). Nomoto et al. analyzed the wearer’s sleep quality and tracked various types of phenomena that affect sleep quality with a wristwatch-based motion sensor worn for a long period [130]. In 2019, Yeom et al. integrated sensors on watches that can analyze sleep apnea and send results in real time to a cell phone [131]. In 2022, Katori et al. analyzed over 100,000 data sets and analyzed the classification of 16 sleep problems [132].

Although there are multiple joints between the wrist and torso in sleep staging and the human body, restoring the overall body posture with the wrist is difficult. In 2019, Trevenen et al. attempted to improve the accuracy of recognition with machine-learning algorithms [135]. In the same year, Walch et al. also attempted to analyze raw acceleration data from Apple Watch to analyze sleep, but the specificity was not satisfactory [136]. In 2022, Ode et al. achieved relatively high sensitivity and specificity by designing an acceleration-based long-term sleep–wake cycle classification and estimation algorithm (ACCEL) based on simple arm acceleration sensor results [137]. It shows that recognition rates can be improved by algorithms when sensors can provide limited information, but it is not feasible to compensate only by algorithms hoping to achieve the effect of more sensors.

In addition to the wrist, the chest is also a common location for placing accelerometers to more effectively reflect the human torso’s motion and detect mechanical signals of respiration and heartbeat (described in detail in Section 4.3). In 2017, Razjouyan et al. also demonstrated that a single chest accelerometer for sleep analysis was closer to the polysomnography results than a wrist sensor [138]. In 2021, Chen et al. built a detection system with temporal memory using long- and short-term memory (LSTM) networks after enriching the sensor data types and also achieved good results. The behavioral sensor on the wrist was able to identify sleep data with 92% accuracy [139].

In addition to the two broad categories mentioned above, the types of sensors for detecting posture and movement are actually very rich in various combinations [140]. Sunderam et al. incorporated MEMS accelerometers in a wearable detector for the head, which aided the training set for partitioning different sleep stages and can potentially be used for neuroprosthetic applications for movement disorders and seizures [141]. Yoshihi et al. achieved a higher accuracy sleep stage analysis based on a single 3D accelerometer of the head [142]. However, the accuracy for sleep stage recognition was only 74.6%. For REM sleep, the accuracy was only 52.7%. Therefore, the wrist and torso are still ideal locations for sensor placement.

When a person moves, vibrations are transmitted to the bedding. So, it is also common to prevent mechanical sensors in bed sheets, pillows, and other locations (Figure 10c). Umetani et al. integrated an IoT system in a comforter that can measure the person’s movement and the bedding to improve sleep quality and prevent accidents during sleep [143]. Xin et al. used a flexible piezoelectric material, polyvinylidene fluoride, to create a flexible piezoelectric film that was placed on a pillow to convert the human force on the pillow into an electrical signal. These methods avoid the occlusion of the quilt in optical methods [144]. Xu et al. integrated a piezoelectric film in the mattress, using PVDF material, with a sensing area of 0.114 m^2^ and a thickness of only 0.28 mm [145]. It can detect motion signals in a large area and instantly alert the elderly in case of abnormal sleep.

### 4.2. Posture Detection

**Figure 11:**
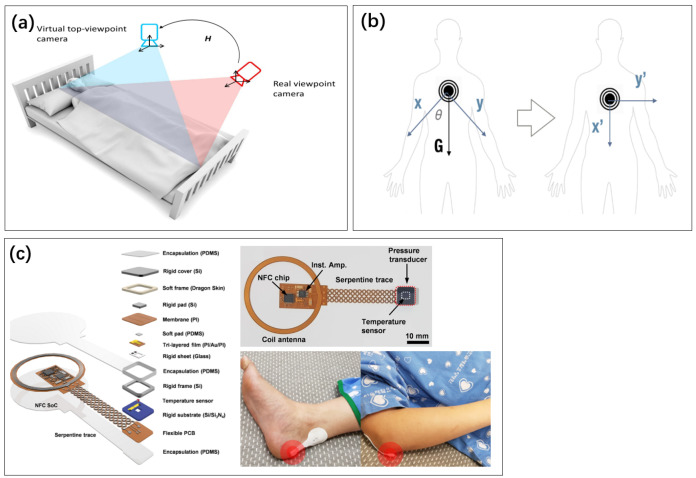
(**a**) Camera images used to analyze sleep posture [5]. Reproduced under the terms of the CC-BY Creative Commons Attribution License, Copyright 2019 by the authors, published by Inst Electrical Electronics Engineers INC. (**b**) The acceleration sensor realizes pose analysis by detecting the direction of gravity [147]. Reproduced under the terms of the CC-BY Creative Commons Attribution License, Copyright 2021 by the authors, published by MDPI. (**c**) Stress sensor stuck to the position vulnerable to pressure when sleeping [148]. Reproduced under the terms of the CC-BY Creative Commons Attribution License, Copyright 2021 by the authors, published by Springer Nature.

Sleep position also has a great impact on sleep quality. A person’s tongue may fall under the influence of gravity when relaxed, obstructing the airway in some positions or under specific conditions, which can lead to snoring or even sleep apnea. Since accelerometers can sense the direction of gravity, many of the studies mentioned in the previous section have detected motion along with pose (Figure 11b) [142]. In 2007, Kishimoto et al. placed accelerometers on the user’s chest to accurately distinguish whether the user was in a supine, prone, or lateral sleeping position compared to sensors on the wrist or lateral sleeping position and could analyze the user’s sleep and wake times based on movement [87]. In 2015, Heenam et al. used patch accelerometers and achieved an average agreement of 99.16% for sleep position assessment [146]. Research on pose detection alone also has important implications in Figure 11.

An infrared camera is an ideal method to analyze human posture. An infrared camera can record human posture without a visible light source, and infrared light has good penetration. Insung et al. used an infrared camera to analyze the effect of sleeping posture improvement on sleep apnea [149]. Cheung et al. used an infrared sensor to monitor the movement of the sleeping elderly and alert the healthcare personnel in times when there is bad activity [150]. Non-contact sleep monitoring based on infrared cameras differed from sleep monitoring devices in the identification of sleep quality by only 4.7%. Infrared array sensors under laboratory conditions are more than 95% accurate in sleep detection [151]. Using infrared sensors together with microwave sensors, the overall accuracy of sleep cycle measurements can be as high as 98% [152].

Force sensors also have many roles in this area. As shown in Figure 11c, a pressure sensor made of a multilayer piezoelectric structure can detect which part of the body is touching the bed and subjected to body gravity [148]. In 2022, Zhang et al. prepared resistive flexible angle sensors using metal foil foils, and the accuracy of flexible wearable sleep posture monitoring devices exceeded 90% [153]. Zhou et al. also achieved sensing of human posture through a bed sheet made of ultra-thin conductive fabric. The sensors can divide the bed into a total of 60 zones and detect in which zones the body’s pressure is located [154].

### 4.3. Sleep Bruxism Detection

During sleep, 8% of the population has reported awareness of tooth grinding [155]. Sleep bruxism also represents the third most frequent parasomnia [155]. People in a state of high mental tension and psychological stress may maintain an excited state of the occlusal muscles at night and experience nocturnal teeth-grinding symptoms [155,156,157,158]. Subjects with obstructive sleep apnea syndrome, loud snorers, subjects with moderate daytime sleepiness, heavy alcohol drinkers, caffeine drinkers, and smokers are at higher risk of reporting sleep bruxism [156]. In 2021, Lee et al. integrated sensors such as accelerometers and gyroscopes in a jaw advancement device used to improve sleep apnea to help monitor the occurrence of sleep apnea and teeth grinding and to improve the effectiveness of related treatment devices [159].

**Figure 12:**
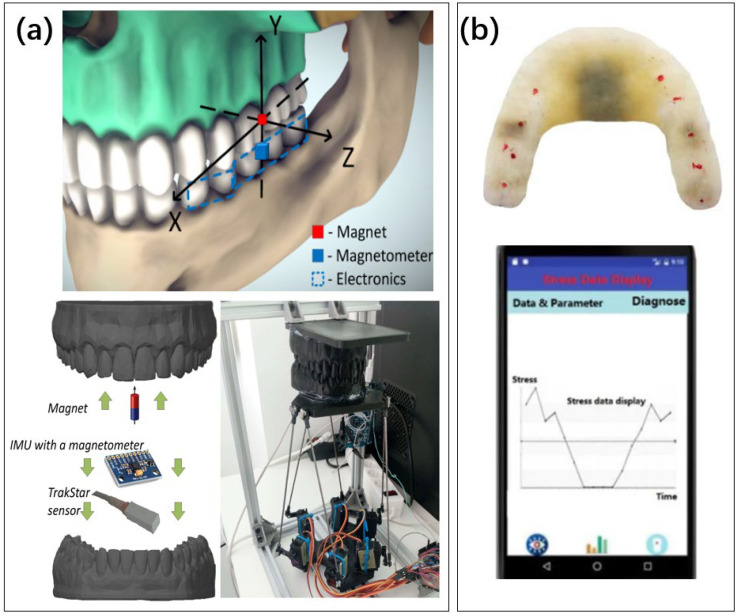
Different methods for detecting bruxism. (**a**) Magnetic field based molar detection [162]. Reproduced under the terms of the CC-BY Creative Commons Attribution License, Copyright 2021 by the authors, published by MDPI. (**b**) The system, composed of strain gauge and microprocessor, is packaged in the tooth socket to form a bite force sensor. The results are sent to the mobile app [164]. Reproduced under the terms of the CC-BY Creative Commons Attribution License, Copyright 2019 by the authors, published by MDPI.

Muscle electrical signals can show the activity of local muscles, but mechanical methods can more truly and directly detect the occlusion method of teeth, as shown in Figure 12. D’Addona et al. measured stresses in the human mouth using a Wheatstone bridge to detect changes in resistance and force output from a miniature strain gauge. When a person undergoes nocturnal teeth grinding, the strain gauges are stressed. The resistance changes and is amplified by the Wheatstone bridge into a voltage signal that can be collected [160]. In 2022, Coimbra et al. used light Bragg grating sensors, encapsulated in a PDMS, to also make wearable pressure sensors that detect different signals from a person biting a splint during an episode of teeth grinding [161]. In 2021, Jucevicius et al. used permanent magnets and a triaxial magnetometer. The magnetic field generated by the permanent magnets at the magnetometer changes when the spatial position relationship between the mandible and maxilla changes, based on which the movement of the jaw joint can be monitored [162]. In 2022, O’Hare et al. used pressure sensors to detect the deformation of the occlusal muscles, which are smaller than myoelectric sensors that are smaller and more accurate in the analysis of occlusal forces. The results of these works are difficult to achieve with myoelectric signals and exemplify the need for mechanical sensors [163].

### 4.4. Mechanical Breath Detection

In addition to the extreme case of sleep apnea, breathing rate and lung capacity are also important physiological information. In 2003, Atanasov et al. found a strong link between nasal cycles and sleep cycles. It is due to the regular circulation of nasal airflow through the nostrils caused by nasal congestion and congestion. In REM sleep, the nasal cycle is synchronized with the sleep cycle [165]. In 2006, Kohler et al. analyzed the effects of different drugs on nocturnal breathing and sleep by detecting airflow and nasal pressure in the right and left nasal passages with three sensors, respectively. Sleep apnea is a common sleep-related disorder that poses a significant threat to the life and health of patients [166]. There have been many studies to detect the occurrence of sleep apnea by heart rate, blood oxygen, and electromyographic signals, which are described in the corresponding subsection. However, the detection of respiratory airflow remains the essential method. Since mechanical signals are important physiological indicators of the respiratory system, relevant sensing is important for both sleep quality monitoring and guidance of treatment [167].

**Figure 13:**
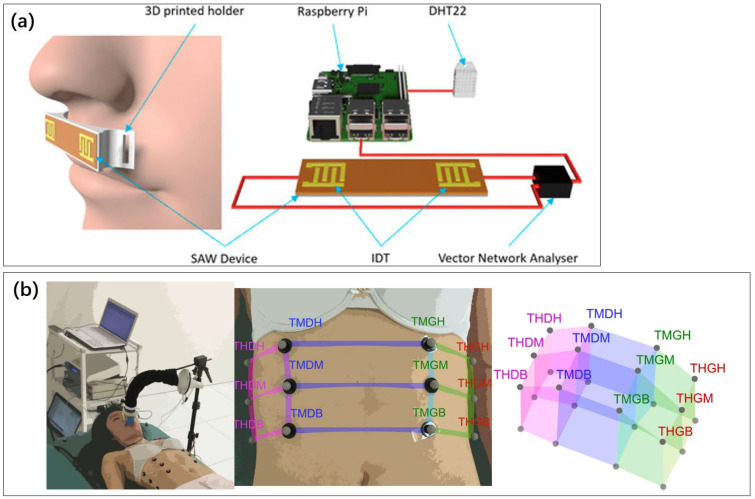
(**a**) Surface acoustic wave sensors [171]. Reproduced under the terms of the CC-BY Creative Commons Attribution License, Copyright 2022 by the authors, published by AIP Publishing. (**b**) A method for measuring thoracic volume using chest marker loci [176]. Reproduced under the terms of the CC-BY Creative Commons Attribution License, Copyright 2022 by the authors, published by Springer Nature.

Teichtahl et al. used thermistors and nasal pressure sensors to analyze whether humans were breathing and to assess sleep apnea well [168]. In 2021, Moshizi et al. prepared nano-complex airflow sensors by growing graphene nanosheets on the surface of PDMS with good sensitivity and linearity [169]. NP thermistor is a common respiratory detection sensor. The detection principle is that the air pressure in the nasal cavity and the temperature at the nostril changes when a person is breathing. In 2018, Jiang et al. combined a respiratory monitor with a motion sensor to more comprehensively screen for sleep apnea [170]. The respiratory sensor designed by Vernon et al. takes advantage of the fact that changes in temperature and humidity in the mouth and nose during human breathing affect the signal of the acoustic surface wave sensor, as shown in Figure 13a [171]. Sleep apnea can be captured sensitively.

In addition, breathing behavior can be reflected by detecting thoracic motion. In 2011, Dehkordi et al. obtained signals about breathing by fixing an acceleration sensor on the sternum. It is also possible to correlate the sleeping position and screen for sleep apnea [172]. Jortberg et al. fixed accelerometers on the chest and measured the respiratory rate (RR) with an average error of 1.84 breaths per minute [173]. In 2020, Yuzer et al. placed accelerometers on the diaphragm, which vibrate a motor on the wristband when the respiratory movement of the diaphragm is detected to stop, stimulating the patient to change the sleeping position until breathing resumes [174]. In 2021, Ghahjaverestan et al. measured the range of motion of the abdominal and thoracic cavities with acceleration and position sensors, respectively, to restore the respiratory signal more precisely [175]. Stubbe et al. fixed 12 markers on the user’s thorax, used an infrared camera to locate the markers, and calculated the volume of the thorax, shown in Figure 13b [176]. This method is known as optoelectronic plethysmography (OEP). The spirometry measured by this method has an error of only 0.4% with the spirometer, and the mechanics sensor provides richer information than the bioelectrical signal that can only measure respiratory rate.

### 4.5. Blood Flow Detection

BP and HR vary with circadian rhythms. Prolonged stress may lead to increased HR during sleep [177]. Although heart rate information can also be obtained from ECG, obtaining heart rate in the scheme without bioelectrical sensing is still meaningful. Moreover, continuous blood pressure detection, including sleep time, is relevant for diagnosing and treating hypertension [178]. Blood pressure also rises at the end of obstructive episodes in patients with sleep apnea, which is undoubtedly dangerous for patients with both obstructive sleep apnea and vascular disease such as hypertension [179]. In 2000, Dimsdale et al. studied the effect of continuous positive airway pressure therapy on blood pressure in patients with obstructive sleep apnea through nocturnal blood pressure monitoring. The significance of nocturnal blood pressure monitoring was demonstrated [180]. However, with an automated device, early detection techniques were based on measuring the patient’s blood pressure every 15 min. A traditional arm band balloon blood pressure detection method was used. Such a method does not allow continuous monitoring, and the sudden working of the air pump and the squeezed arm can affect the patient’s sleep quality.

In 2006, Kaniusas et al. used a magnetoelastic skin curvature sensor to measure carotid blood pressure, enabling continuous blood pressure measurement at night. However, this method has limited accuracy, with a correlation coefficient of less than 0.9 between measured and reference values [181]. Such stress-based blood pressure sensors can cause discomfort from local compression when worn for long periods, both in the fingers and in the arm. Muscle movement and postural changes can alter the mechanical environment to affect measurements [182]. A more non-sensitive blood pressure monitoring that people can wear for long periods at night and does not interfere with their sleep quality is the only truly usable technology for nighttime blood pressure monitoring.

**Figure 14:**
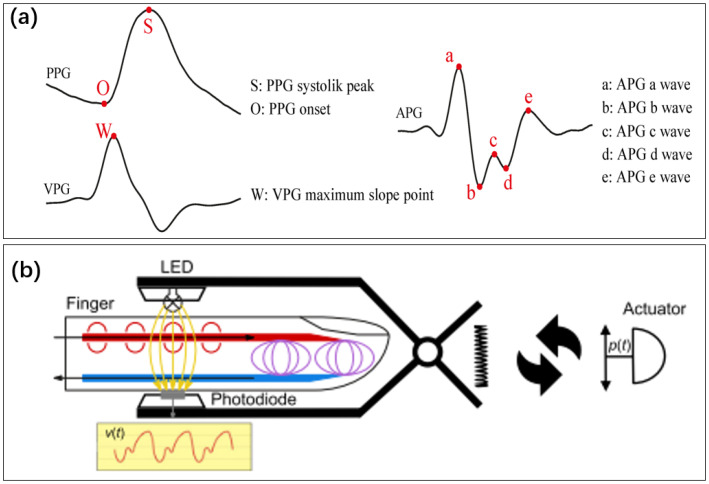
(**a**) Graphical definition of fiducial points detected from photoplethysmogram (PPG), velocity plethysmogram (VPG), and acceleration plethysmogram (APG) signals [188]. Reproduced under the terms of the CC-BY Creative Commons Attribution License, Copyright 2022 by the authors, published by MDPI. (**b**) Optical sensor for detecting blood flow in fingers [189]. Reproduced under the terms of the CC-BY Creative Commons Attribution License, Copyright 2021 by the authors, published by Springer Nature.

Heart rate and blood flow sensing based on optical signals have the advantages of being non-invasive and having low wearing requirements. Photodensitometry, a technique widely used to monitor blood volume changes according to the Lambert–Beer law, is more suitable for home use [183]. This can be used for heart rate monitoring, allowing sleep staging. A type of blood volume sensor—a technology known as photoplethysmography (PPG)—is also widely used [184]. From this, the heart rate and other physiological parameters can be extracted to inform about user activity, fitness, sleep, and health, as shown in Figure 14. However, sleep staging based solely on heart rate measured by PPG waves has a low classification accuracy of between 55% and 78%. Sleep staging combining exercise and heart rate also has an accuracy of 78.2% and cannot replace sleep polysomnography [185,186]. The recognition accuracy can be improved to more than 93% by machine-learning techniques such as data augmentation and convolutional neural networks [187].

Although less accurate independently as a basis for sleep staging, several works have reported the detection of blood flow under the skin at night by light sensing and recording the time of pulse passage (PTT), which can restore blood pressure information by biomechanical models. Blood pressure can be calculated by measuring the time the pulse wave travels through the blood vessels [178]. Measurements can be made using the difference in the speed of propagation of the ECG signal and the pulse wave signal. Shahrbabaki et al., based on fixing sensors at the fingertips [190], and Kireev et al., based on electronic skin [117], respectively, achieved reliable nocturnal measuring. Zadi et al. performed the error of blood pressure measurement by PTT in different states, and the mean value of the model residuals was considered to be less than 3.2 mm Hg during both normal breathing and breath-holding maneuvers [191]. Krefting, based on this technique, successfully observed elevated blood pressure due to different postures at night [192]. Carek et al. integrated sensors in underpants, detected the signal when blood pulses flowed through the legs, and correlated with cuffed sensors [193]. PTT-based blood pressure functions have been integrated into smart watches [194].

PTT measurements can also be performed by two mechanical sensors located in different parts of the body. Thin-film piezoelectric sensors give the possibility of wearability of this sensing. Xin et al. achieved blood pressure monitoring with a standard deviation of only 1.7 mm Hg using two flexible sensors made of PVDF piezoelectric material. Resonant amplifier circuits were designed, too [195]. PTT measurements were also implemented by Fan et al. using textile electronics [196].

Mechanics signals can also be used for heartbeat detection. Heart rate measurements were performed by Sanchez et al. by collecting vibration signals in the chest cavity [197]. Xin et al. collected signals from pulse vibrations using thin films made of flexible piezoelectric materials [144]. The ultra-thin conductive textile bed sheet made by Zhou et al. has a wide operating frequency bandwidth range of 0 Hz to 40 Hz, good mechanical durability, and washability [154]. Its high sensitivity allows the sensor to obtain heart rate measurements with an error of only 1.33% without wearing any wearable device specifically. However, HR detection by mechanical methods is not common because optical sensors have been able to monitor HR effectively and accurately.

In the last method, Van et al. monitored the heart rate of users based on an infrared camera, using three different frequencies of infrared sensors and a broad spectrum of infrared light sources to achieve transmission of the occlusion [198]. The heart rate detection accuracy was 92%. In addition, because the camera does not touch the body at all, it is not disconnected by the person’s movement, as is the case with ECG. After the person’s posture changes, the camera can also be positioned again in time and continue to measure heart rate.

### 4.6. Acoustic Detection

The human body has many mechano-acoustic (MA) signals [199]. Snoring is a common phenomenon in sleep, which is caused by the poor ventilation of the airway and has a significant impact on the quality of sleep and in some cases may lead to sleep apnea or even asphyxia. Dafna et al. analyzed the sleep of patients with obstructive sleep apnea by recording their sleep sounds [200]. The accuracy of snoring frequency monitoring can be improved from 81% to 89% by presetting age and gender information [201].

Human sleep is also susceptible to the effects of environmental sounds. Many sleep monitoring systems now integrate ambient sound acquisition to help people analyze the causes of poor sleep. Some sounds may prevent a person from falling asleep, while white noise has shown sleep-aiding effects in many studies [202]. Chen et al. integrated ambient sound sensors into the sleep monitoring system, which automatically plays white noise to mask the noise affecting people’s sleep when ambient noise is detected, and the body sensors in the system monitor sleep quality in real time [203].

If just collecting the sound in the room, perhaps no special equipment is needed. Snoring signals have strong penetration, so sensing these can often be implemented directly with the help of a smartphone [202]. Xin et al. prepared acoustic sensors using flexible piezoelectric films to detect snoring, but there is no significant advantage over existing integrated microphones [144,204].

However, in some cases, acoustic sensors close to the skin can give more in vivo information. Ghahjaverestan et al. analyzed respiratory airflow by measuring the sound of tracheal airflow through a microphone close to the skin and used it for the assessment of sleep apnea [175]. Li et al. designed a sensor that can be attached to the chest [199]. It is very sensitive to snoring, and the measured frequency information can be interpreted by human anatomy.

### 4.7. Other Mechanical Detection

**Figure 15:**
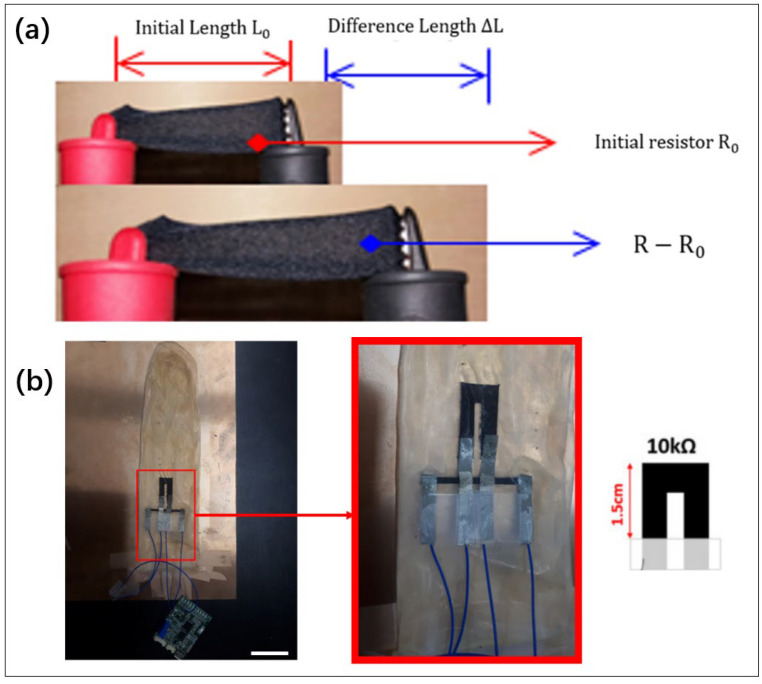
(**a**) A film that detects strain. (**b**) A combination of strain sensors that detects changes in penis length and diameter [210]. Reproduced under the terms of the CC-BY Creative Commons Attribution License, Copyright 2021 by the authors, published by MDPI.

In men, penile engorgement and erection occur during REM sleep and are important physiological phenomena accompanying the sleep cycle [205]. There is also an association between erectile dysfunction and sleep disorders [206,207]. In 2021, Krkovich et al. enabled monitoring of erections during sleep by recording the diameter of the user’s penis. The results obtained made it possible to determine reference values for qualitative and quantitative indicators of PNT in healthy male volunteers [208]. In 2022, Edgar proposed several forms of sensors that could be used to monitor nocturnal sleep erections: a penile arterial pulse for measuring plethysmograph, a displacement sensor to measure axial length, a strain gauge to measure radial stiffness and circumference, and a temperature sensor to measure skin and cavernosal temperature [209]. In 2022, Heo et al. used an electronic fabric strain sensor to replace the bulky and heavy Rigiscan device in Figure 15. By immobilizing carbon nanotubes on the fabric, resistive length, perimeter, and curvature measurements were achieved. The result shows a 1.44% error rate and a cavity radius of 110 to 300.

Rapid eye movements are a hallmark feature of REM sleep and can reflect how neurologically active a person is under that stage of sleep. Although, the occlusion of the eyelids makes eye movements less easily observable, so the EOG in the previous section is a more common way to detect eye movements. Many studies have reported direct mechanical measurements of eye movements. In 2020, Wu et al. designed a smart eye mask [211]. The hydrogel sensor was integrated with a sleep mask for real-time monitoring of human sleep. Compared to the sleep recorded using a popular sleep monitoring mobile app that measures sleep only based on body movements and voices, the sleeping process measured using the smart sleep mask shows much higher reliability for the recognition of REM sleep. In 2021, Dang et al. used an infrared optical sensor integrated with an eyecup to detect eyelid-surface-shaped edges caused by eye movements and used an array of four sensors to each detecting motion in two vertical degrees of freedom [212].

The last mechanical signal is the intraocular pressure (IOP). Continuous monitoring of IOP, especially during sleep, remains a great challenge for glaucoma care. Zhang et al. designed contact lenses with integrated strain sensors and induction coils that allow continuous IOP monitoring at night but with some discomfort [213]. However, very mature, formally usable studies have not been reported. The important difficulties are the passive wireless devices and the lack of oxygen caused by wearing the device all night.

### 4.8. Summary

The biomechanical signal monitoring techniques presented in this section are often related to specific behaviors, for example, movement, teeth grinding, restless legs, erections, etc., but also the monitoring of physiological phenomena such as respiration and heartbeat. Since there are major differences in the objects and purposes of detection, it is difficult to compare them at the methodological level from a unified dimension. Table 2 of this section is more like a summary. Sleep stage is an important indicator of sleep monitoring, which also appears in many studies and is therefore listed separately.

## 5. Biochemical Signal Detection

**Figure 16:**
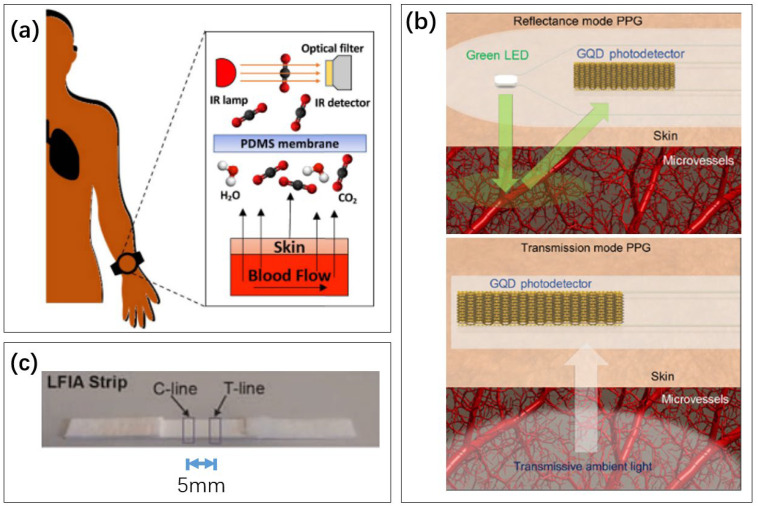
Examples of three biochemical detection methods in this section. (**a**) The concentration of carbon dioxide through the skin is detected by a gas sensor [218]. Reproduced under the terms of the CC-BY Creative Commons Attribution License, Copyright 2021 by the authors, published by Inst Electrical Electronics Engineers INC. (**b**) A blood oxygen sensor based on the detection of reflected and transmitted light [219]. The absorption of light by hemoglobin is different when the blood oxygen level is different. Reproduced under the terms of the CC-BY Creative Commons Attribution License, Copyright 2019 by the authors, published by American Association for the Advancement of Science. (**c**) Immunofluorescence test paper for detecting cortisol in saliva [220]. Reproduced with permission, Copyright 2014 Elsevier B.V.

Biochemical tests have important applications in medicine. The physiological activities of the human body, such as immunity, endocrinology, and cellular metabolism, can be realized to a large extent by the detection of the concentration of relevant substances in the body. With the maturity of non-invasive testing technology, wearable health testing devices can also realize chemical sensing. For example, blood drug concentration detection using electrochemistry [215], wearable continuous glucose monitoring [216], etc. These devices can also be used while sleeping, and there have been reports about them [217]. In this paper, we focus on three biochemical assays that are closely related to sleep, shown in Figure 16.

### 5.1. O_2_ Level Detection

Blood oxygenation is a common physiological indicator. In 2013, Elizur et al. identified the effects of hypoxemia on glucose metabolism during REM sleep [221]. In 2014, the patterns of brain tissue oxygen content changes in adults and adolescents during different sleep stages were revealed by NIRS [222]. In 2022, Elmenhorst et al. used blood oxygen sensors to analyze the sleep quality of long-haul flight crews at high altitudes and investigated the effect of a hypoxic environment on sleep at high altitudes, which has important implications for civil aviation safety [223,224,225,226].

The oxygen content of the different parts of human vasculature is different. Easily measured and well-referenced is the percutaneous arterial oxygen saturation (SpO_2_). Cakmak et al. detected obstructive sleep apnea with the help of an optical blood oxygen sensing device. This relies on the different absorption rates of light by hemoglobin in the human body before and after binding oxygen, which in turn monitors the oxygen content within the blood through a reflected light sensor [227]. The finger is rich in capillaries, which is a common location for blood oxygen detection [226]. The dual-channel continuous oxygen saturation sensor designed by Zhang et al. explored different types of fingers as well as different wearing positions. It ended up with a root-mean-square error of only 1.8 [228]. The correlation coefficient tested by Tran et al. reached 0.93, with a 95% agreement limit of ±2.5% [229].

**Figure 17:**
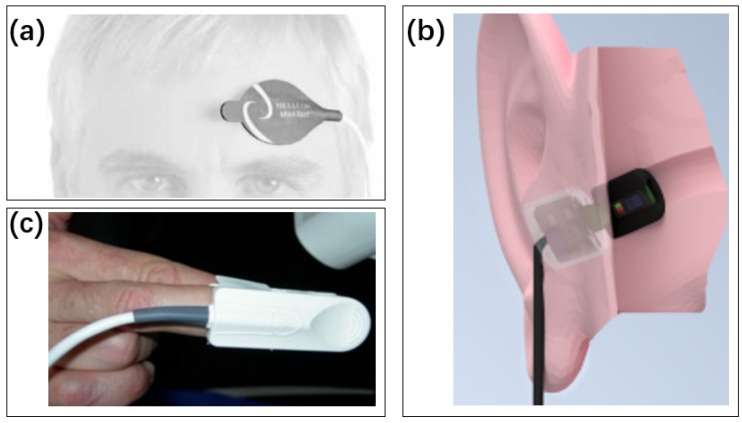
Positioning of the pulse oximeter sensor. (**a**) Forehead [224]. Reproduced with permission, Copyright 2004 Blackwell Publishing Ltd. (**b**) In ear [225]. Reproduced under the terms of the CC-BY Creative Commons Attribution License, Copyright 2020 by the authors, published by MDPI. (**c**) Finger [226]. Reproduced under the terms of the CC-BY Creative Commons Attribution License, Copyright 2021 by the authors, published by MDPI.

Because optical signals can easily detect oxygen levels in the blood at any capillary, various types of wearable sensing devices can be used. Earlobe sensors [230] and ear canal sensors [231] can be developed without being limited to common locations such as the wrist, as shown in Figure 17. Among them, brain tissue oxygen saturation is very important for the quality of sleep, and Metz et al. designed measurement of this using near-infrared spectroscopy to detect the oxygen saturation of brain tissue before and after human sleep [222,232], since the oxygen content of human arterial and venous vessels is not the same, and the veins at the arms may interfere with the results. Capillary-based monitoring in areas such as between the fingers, which is 2–3% higher than the armed vessel oxygen content test, is more suitable as a measure of sleep apnea [233]. Nabavi et al. used intraoral photoplethysmography and showed more than 96% accuracy in estimating physiological characteristics such as SpO_2_ compared to conventional monitoring techniques [234].

In 2021, Van et al. used a broad-spectrum infrared light source to enhance the infrared signal and used three infrared cameras with different frequencies to measure the reflected light signal and calculate the blood oxygen concentration. Based on this, non-contact infrared measurements were achieved, avoiding the detachment of the sensing device from the body due to motion. The non-contact oximeter estimated blood oxygen values with an 89% time error within four 4% [198].

### 5.2. CO_2_ Level Detection

The carbon dioxide level is another important test. Insufficient sleep breathing at night may cause hypercapnia, leading to respiratory failure. This process occurs mainly due to increased carbon dioxide levels in blood vessels, such as arteries, due to hypoventilation, in relation to the person’s height, body mass index, and the degree of obstruction of the upper airway [235]. In cardiac patients, the partial pressure of carbon dioxide at night alters their pathophysiology during the day and night.

**Figure 18:**
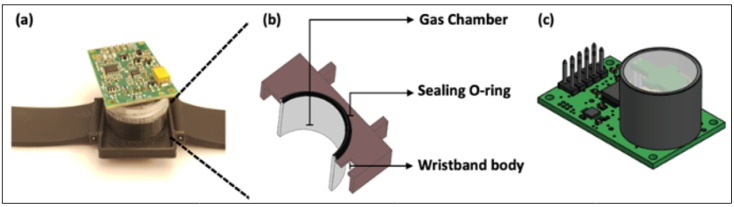
Wearable transcutaneous CO_2_ monitor based on miniaturized non-dispersive infrared sensor. (**a**) Image of the integrated wristband, ø20.9 mm × 18.1 mm. (**b**) The zoom-in shows cross-section view of the gas chamber, sealing O-ring, and wristband body. (**c**) Miniaturized Cozir NDIR CO_2_ sensor [218]. Reproduced under the terms of the CC-BY Creative Commons Attribution License, Copyright 2021 by the authors, published by Inst Electrical Electronics Engineers INC.

Ramos et al. measured carbon dioxide levels, volatile organic compounds (VOCs), and air temperature in indoor environments using low-cost gas sensors [236]. Rauhala et al. used electromechanical film sensors, a flexible material that can analyze carbon dioxide concentrations in blood vessels on the skin’s surface through differences in electrical signals of the skin’s surface and analyze increased carbon dioxide concentrations due to sleep apnea [237]. Kang et al. used gas sensors to measure the concentration of breathing gases in human bodies [238]. Chhajed et al. used an earlobe carbon dioxide sensor to monitor nocturnal carbon dioxide concentrations and monitored the effectiveness of positive pressure ventilation for chronic hypercapnia. This took advantage of the fact that small carbon dioxide molecules have high tissue solubility and can diffuse rapidly through the skin [239]. Tipparaju et al. improved the accuracy of a transdermal continuous carbon dioxide sensor by solving humidity interference with a miniature non-dispersive sensor through a hydrophobic membrane, as shown in Figure 18.

### 5.3. Hormone Detection

The human body has many hormones that play a regulatory role in the sleep process. For example, melatonin plays an important role in regulating circadian rhythms in humans. Through feedback to light, the accumulation of melatonin in the body will make people sleepy [240,241,242]. Melatonin is also now available to treat some insomnias [243]. In addition, diseases such as adrenal hyperplasia or a prolonged state of emergency may lead to an overproduction of hormones such as adrenal hormone, norepinephrine, and adrenocortical. These hormones can put a person in a hyperactive state and cause difficulty falling asleep, poor sleep quality, and easy awakening [244,245].

Multi-hormones can be detected in human saliva through secretory glands and thus enable non-invasive sensing. Previously, the level of hormone detection in non-invasive samples was mainly performed by sending the samples to the laboratory and detecting them by fluorescent probe method without real time. Massey et al. prepared a non-invasive body fluid sensor based on EG-FET to monitor the cortisol hormone concentration in saliva samples. The detectable cortisol concentration range is currently identified as 27.3 pM–27.3 μM [246]. Shahub et al., on a nanoporous matrix by electrochemical impedance spectroscopy, measured cortisol concentrations in sweat with 100% accuracy and 0% false negatives, with a dynamic range of 8–140 ng/mL [247].

Sensor research at the hormone level is less compared to the rest of the field, but with the maturation of wearable chemical sensor technology, the future promises to provide more health information at the secretory system and drug therapy level.

### 5.4. Prospect of Biochemical Detections

Among the studies related to sleep detection, biochemical detection has been significantly less studied than in the previous two sections. This is probably because chemical sensors tend to be more complex. However, there is no substitute for the importance of biochemical signals in sleep monitoring. Melatonin is the most commonly used medication for insomnia, and caffeine intake is the most common method used when people want to stay awake. Biochemical methods are commonly used to treat sleep-related problems. Biochemical-based tests can guide individualized treatment.

By tracking cortisol concentrations, Dornbierer et al. demonstrated that pulsed-release caffeine could help people suffering from insomniac sleep inertia to wake up from sleep faster [248]. In addition, Julia et al. demonstrated that caffeine concentrations in the body could be detected in sweat from the fingertips after coffee consumption [249]. Akiyo et al. used optical methods to measure ATP concentrations in the brains of mice and observed fluctuations in the intoxicated sleep–wake process [250]. This allows analysis of brain activity in terms of energy metabolism and has important implications for some abnormalities in the neurological causes of the brain during sleep.

**Figure 19:**
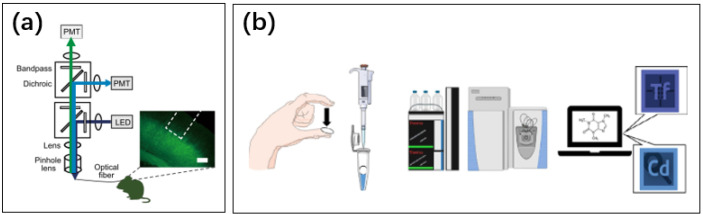
(**a**) Measurement of ATP concentration in mouse brain using optical methods [250]. Reproduced under the terms of the CC-BY Creative Commons Attribution License, Copyright 2021 by the authors, published by Springer Nature. (**b**) Detection of caffeine concentration in finger sweat in the laboratory [249]. Neither method is available for people to use at home by themselves. Reproduced under the terms of the CC-BY Creative Commons Attribution License, Copyright 2020 by the authors, published by Springer Nature.

The related study shown in Figure 19 may not be designed for at-home sleep monitoring, so it is not yet available for people to use daily. This is a valuable direction for future research. With the development of relevant biochemical detection technology, doctors may be able to prescribe more personalized prescriptions or even automatically adjust the use of drugs based on the hormone levels detected in the user’s body each night.

## 6. Multi-Signal Sleep Monitoring

**Figure 20:**
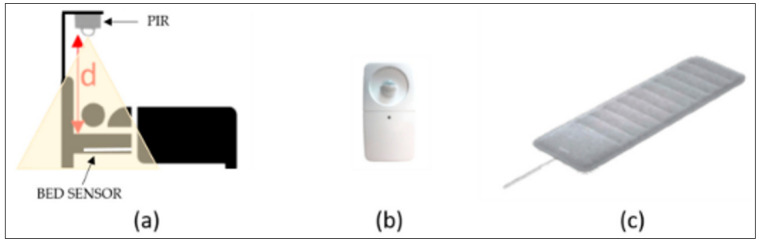
Examples of multiple signals. (**a**) A two-channel monitoring system. (**b**) PIR sensor; (**c**) Nokia sleep bed sensor [255]. Reproduced under the terms of the CC-BY Creative Commons Attribution License, Copyright 2019 by the authors, published by MDPI.

Some studies use single signal sensors with algorithms that achieve certain analysis functions. Nowadays, smart detection devices in the market are mainly based on smartwatches and smart bracelets, and the measurement of total sleep time is accurate with good sensor quality. Still, the results of complex analysis, including measuring different sleep stages, are not yet satisfactory [251,252,253,254]. Current consumer sleep-tracking technologies may not be mature in diagnosing sleep disorders, and more multi-signal sensors have much room for research. For example, Figure 20 shows the combined use of an infrared camera and bed sensor.

### 6.1. Multi-Signal, Single Physiological Information

The first category uses multiple sensors of different types to jointly analyze a particular physiological activity, such as REM sleep detection, sleep apnea detection, etc. Models that analyze sleep based on individual sensor data may vary with the underlying conditions of different individuals. For example, the EEG sensor and model designed by Sharma et al. achieved an accuracy of 83% in the sleep stage classification test for healthy individuals [256]. However, the same method yielded an accuracy of only 72% for patients with an REM disorder. Different types of sleep-related disorders have an impact on the results. Deep-learning methods have limited effectiveness in solving this problem [35]. In this case, introducing the rest of the sensors for auxiliary classification is often necessary. Another method is to use multiple signals for sleep stage classification.

Sleep apnea, sleep stage division, and other physiologies with multiple signal presentations are the most common. A relatively simple monitoring such as respiratory rate can also be boosted with a multi-channel signal. Combining different broad categories of signals helps better complement each other but also adds more cost and invariance to the use. For the identification of sleep apnea, the best studies have increased the accuracy to 100%. Respiratory rate monitoring can also have an error of less than one per minute. The complete information is provided in Table 3. The future direction of this type of research will be comfortable to wear. In addition, there is still room for improvement in the truth rate of some behavioral monitoring.

### 6.2. Single Sensor, Multiple Physiological Information

The second category is the implementation of different tests based on single-sensor hardware with different usages. Sometimes the sensor’s response may be related to several different mechanical, biochemical, etc., signals. For example, the PPG sensor can measure blood pressure and heart rate separately when measuring different values. Another example is the limb acceleration signal, which is a superposition of multiple signals such as posture, movement, and respiration and thus can be interpreted with multiple information. So, some studies, although based on different physiological signals, may ultimately be achieved using the same hardware in different ways.

The accuracy of heart rate and blood oxygen monitored by a single sensor has been reported in many studies. However, no new protocols have emerged from such studies, and several major protocols are relatively well-established. More accurate measurements or more comprehensive analyses require multi-bed sensor combinations.

The paper is divided into sections according to physiological signal categories, and this subsection adds results that are somewhat classified from a sensor perspective. Achieving multiple monitoring through a single sensor can significantly reduce costs. The complete information is provided in Table 4. Here, we can see studies that use a single sensor to acquire multiple signals and enhance the correlation between different signals.

### 6.3. Integrated Sleep Monitoring

The third category is using multi-signal sensors for a comprehensive sleep quality analysis. It is similar to a PSG system for home use.

The ideal product provides professional PSG monitoring under in-home conditions. It includes accurate sleep stage classification and disease diagnosis. It should also be easy to use, inexpensive, and not interfere with sleep. There is much room for improvement in integrated sensing solutions. Some existing commercial solutions appear in the table, with advances in the laboratory.

The combinations of signals and sensing modalities are very diverse and difficult to exhaust. The ability to achieve the best-integrated sleep monitoring under home conditions with the lowest cost and most user-friendly combination of sensors is the core research goal. Many researchers have investigated how to improve this combination. The complete information is provided in Table 5. In the table, there is an example of the conclusion that some techniques can detect anomalies earlier than others. Many studies have also been conducted on special populations or commercial devices.

### 6.4. Summary

In summary, multi-channel monitoring showed many better results than single channels. EEG and ECG are information-rich sensors, but the combination is still not comparable to professional polysomnography monitoring. More sensing is useful.

However, sleep monitoring with good results is not a simple combination of sensors in the previous three sections. Random combinations of unrelated sensors or multiple interpretations of individual sensor data may improve classification but with limited results.

Good combinations of sensors often come from a particular physiological phenomenon or object to be measured, with multiple different facets of performance. Multiple interpretations of data are common since multiple effects inherently modulate a given physiological signal. In this context, the construction of multi-channel detection devices and the development of related algorithms make sense.

## 7. Conclusions and Discussion

Sleep monitoring is important in an era when people are increasingly concerned about their health. Sleep is also related to the function of many body systems, diseases, and health conditions. In addition to the most basic sleep duration and quality records, new tests can help people find the causes that affect sleep. Sleep apnea, sleep grinding, restless legs syndrome, and other disorders affecting sleep have been reported in many tests. Techniques such as blood pressure, environmental, and endocrine monitoring can help give people more insight into the causes of their poor sleep. However, many other triggers of poor sleep still need to be tested only in the hospital, such as white matter hyperplasia of the brain [266].

Many wearable devices and bedding for sleep monitoring have been commercialized. However, there is room for further improvement in their accuracy and reliability. The current research, taking PSG as the comparison standard, still fails to reach the accuracy of the clinical level. In addition, the detection effects become even worse when seeking senseless use. The balance between performance, versatility, cost, and ease of use needs to be found to suit the consumer. Professional polysomnography monitoring in the clinical setting also remains difficult to replace.

The development of sleep monitoring technology is the miniaturization, wearability, and senselessness of existing sensors. On the other hand, there is also a need for better models and algorithms to help people improve their health. The development of artificial intelligence has brought greater possibilities for the back-end algorithm of the sensor, but this does not replace the improvement of the sensor itself. There are also many reports of cutting-edge laboratory results, including hormone testing. Future sleep monitoring in home and clinical settings is expected to expand its capabilities further.

## Figures and Tables

**Table 1:** Table of electrodes for bioelectrical monitoring.

Type	Contact Resistance	Electrode Size	Correlation	Feature	Ref.
Wet/semi-dry Electrode	1.5–130 kΩ	mm–cm	60–100%	Most commonly used in clinical practice.	[56,75,121]
Dry electrode	2.5 kΩ–5 MΩ	mm–cm	60–98%	Easiest to use.	[121,122]
Conductive fabrics	3.4 kΩ–34 kΩ	cm–dm	50–95.6%	The maximum contact resistance min. The same experience as regular eye masks and pillowcases.	[62,95,104,121,123]
Microneedle array	14.16–378.18 kΩ cm^2^	mm–cm	60–95%	Minimum contact resistance of in vitro electrodes.	[102,121]
Implantable electrodes	100 Ω–34 kΩ	μm	/	Best signal quality. Surgery is required.	[80,81,82,83,124]
Contact lens electrodes	/	mm–cm	/	Dedicated to ERG	[114]

**Table 2:** Table of biomechanical signal monitoring.

Methods/Technology	Monitoring Objects	Sleep Stage	Accuracy (Error)	Feature	Ref.
Record the usage time of cell phone keyboard	/	Sleep–awake	(9.83 + 5.40 min)	Related to cell phone usage habits. Does not require any new devices	[125]
Wristband accelerometers	7 types of insomnia	Sleep–awake	/	/	[132]
Wrist accelerometer	/	NREMS	96.90%	Accuracy for REMS is low, comparing different classification algorithms	[135]
Wrist accelerometer (apple watch)	/	3 stages	72%	Exercise alone is better than HR alone. The combination can be improved to a certain extent	[136]
Wrist accelerometer	/	Sleep–awake	91.71%	The algorithm takes into account the tic behavior	[137]
Wrist accelerometer	/	Sleep–awake	95.80%	Use commercial products, add HR analysis	[139]
Chest acceleration	Sleep position	/	99.16%	/	[146]
Chest acceleration	/	Sleep–awake	85.80%	6% higher than wrist under the same conditions	[138]
Wrist and chest orientation sensors	Sleep position		95%	The combination of different positions was compared	[140]
Head accelerometer	/	3 stages	(2.0–5.2%) with EEG	Help EEG improve accuracy	[141]
Head accelerometer	/	4 stages	74.6%		[142]
Quilt accelerometer	Accidental falls	/	/	There is no need to wear	[143]
Smart watches	Posture, movement, sound	/	87–98%	/	[214]
Piezoelectric film mattress	Abnormal sleep in the elderly	/	/	/	[142]
Chest and wrist accelerometers	Sleep position	/	85%	/	[147]
Infrared camera	In bed state	/	99.80%	Non-contact	[150]
Infrared array	Sleep position	/	95%	Non-contact	[151]
Microwave sensor, infrared sensor	/	4 stages	98.65% + 0.05%	Non-contact	[152]
Capacitive, accelerometer	Restless legs syndrome	Sleep–awake	83.72%	/	[129]
Ultra-thin smart textiles	Sleep position	/	/	Non-contact	[154]
Intraoral accelerometer	AS, Sleep position	/	/	/	[159]
Intraoral magnetic sensors	Teeth grinding	/	(0.260 + 0.004 mm)	/	[162]
Intraoral pressure sensor	Teeth grinding	/	82.20%	Close to EMG results	[163]
Nasal pressure and oro-nasal thermal sensor	Respiratory events	/	Up to 94%	/	[168]
Airflow, activity	OSAS	/	96.50%	/	[170]
Chest acceleration	Spirometry, RR	/	−1.50%	/	[172]
Chest acceleration	RR	/	(0.26 bpm)	/	[173]
Accelerometer near the diaphragm	SA	/	100%	Vibrations stimulate the body to change posture	[174]
Tracheal sound sensor	Breath airflow	/	/	/	[175]
OEP	RR	/	−0.40%	/	[176]
Skin curvature sensor	BP	/	(4 mmHg)	Poor correlation	[181]
PPG	BP, HR	Sleep–awake	Up to 93%	/	[185,187]
PTT	BP	/	(3.2 mmHg)	/	[117,190,191]
Chest acceleration	HR	/	95%	/	[197]
Infrared camera	HR	/	92%	Non-contact	[198]
Microphone		Sleep–awake	82.10%	Non-contact	[200]
Microphone	Snoring	/	89%	Non-contact	[201]
Electronic fabric strain sensors	Nocturnal erection	/	(1.44%)	/	[209]
Sensors on contact lenses	Eye pressure	/	/	Can warn of high eye pressure problems during sleep	[213]

**Table 3:** Table for analyzing single subject based on multiple sensors.

Objectives	Sensors	Accuracy (Error)	Feature	Ref.
Sleep Apnea	Nasal airflow sensor, body activity sensor, SpO_2_ sensor	96.5%	Specificity of 100%	[170]
Sleep Apnea	Utilizing thermocouple; pulse oximeter	100%	Wireless data sharing	[227]
SRBD	ECG, microphone	89%		[257]
Sleep Stages	EEG EOG	89.2%	The recognition rate of non-REM sleep stage 1 is low	[258]
Sleep Stages	3-axis accelerometers, respiratory acoustic sensor, four infrared optical sensors	/	Integrated into the eye mask	[212]
Breathing rate	Bioimpedance sensor, temperature sensor	(0.71 bpm)	Effectively in different postures and dynamic environments	[259]
Grinding	Masseter pressure sensor, masseter EMG	82.8%	Pressure sensors are less accurate than combined sensing	[163]
Restless Leg Syndrome	Capacitive sensors; six-axis inertial measurement sensor	93.65%	Effectively improve diagnosis rates	[129]
Ventricular Bigeminy	ECG, microphone	/	The delay was reduced by up to 88%	[93]

**Table 4:** Table for multi-signal detection based on a single sensor.

Sensor	Outputs	Accuracy (Error)	Feature	Ref.
Infrared camera	Pulse rate, respiratory rate, blood oxygen	92%	No contact	[198]
Optical Blood Oximeter	Pulse rate, blood oxygen	/	Vibration makes people adjust their posture when breathing is not good	[260]
Optical Blood Oximeter	Pulse rate, blood oxygen	99%		[227]
Intraoral photoplethysmography	Pulse rate, respiration rate, respiration pattern, blood oxygen	96%		[234]
Acoustic sensor	Pulse rate, respiration rate	(2.6–3.9 bpm)	Mild with anatomical structure-based interpretation	[199]
Piezoelectric film	Movement, pulse rate, respiratory rate, blood pressure	(3 mm Hg)		[195]
Conductive textile	Posture, pulse rate, sleep apnea	(1.33%)	Can be washed repeatedly	[154]
Textile electronics	Pulse rate, respiration rate, PTT, SAS	/	Can be fixed in any position, washable	[196]

**Table 5:** Table of integrated sleep monitoring.

Sensors	Output	Indicators	Feature	Ref.
Infrared depth sensor, camera, four-microphone array	Sleep quality analysis	/	Automatic play of white noise to improve sleep quality	[203]
Acceleration sensor, temperature sensor, humidity sensor	The movement of the person and bedding	/	No need to wear wearable devices	[143]
Passive infrared sensor, bed sensor (Nokia sleep bed sensor)	Sleep latency, sleep interruptions, time to wake, sleep efficiency	4.7% robust statistic confidence	Sleep quality can be effectively assessed	[255]
Galaxy Watch (PSG sensor, PPG sensor, 3-axic accelerometer)	Sleep stages, epoch-by-epoch respiratory events classification, snore events classification, blood oxygen	77% accuracy in sleep stages prediction, 80% accuracy in epoch-by-epoch respiratory events classification, 60% accuracy in snore events classification 70% accuracy in SpO_2_ level classification	Commercial integrated wearable devices	[261]
ECG, accelerometry,	Heart rate and 5 ECG characteristics, posture, sleep quality	/	Cardiac changes start earlier and last longer than movement	[262]
Single-channel EEG; nasal pressure transducer and thermistor; thoracic and abdominal respiratory inductance plethysmograph belts; pulse oximetry; EMG	Sleep-disordered breathing and periodic leg movements	Failure rate was reduced to 19%	/	[263]
EDA; ACC; skin temperature sensor	Sleep/wake; high/low sleep quality	92.2% accuracy of sleep–wake, 61.51% accuracy of low sleep quality	/	[264]
Accelerometer, gyroscope, orientation sensor; microphone; ambient light sensor	Sleep posture and habits, environment, sleep quality	98% accuracy of event detection	Identify causes for sleep problems compared to prior work	[214]
MEMS triaxial accelerometer, pressure sensor	Vital signs, snore events, and sleep stages	97.2% accuracy of snoring, 95.1% accuracy of sleep stage	/	[265]

## Data Availability

Not applicable.
